# The Predicted Structure of *S. cerevisiae* Ssp1 Reveals Parallel Evolution in the Pil1 BAR Domain Family Proteins of *Ascomycetes*

**DOI:** 10.3390/jof11090661

**Published:** 2025-09-09

**Authors:** Yasuyuki Suda, Aaron M. Neiman

**Affiliations:** 1 Laboratory of Molecular Cell Biology, Faculty of Medicine, University of Tsukuba, 1-1-1 Tennodai, Tsukuba 305-8575, Ibaraki, Japan; ysuda@md.tsukuba.ac.jp; 2 Department of Biochemistry and Cell Biology, Stony Brook University, Stony Brook, NY 11794-5215, USA

**Keywords:** sporulation, BAR domain, gene family, evolution

## Abstract

BAR domains are a superfamily of widely conserved membrane binding motifs. In fungi, Pil1 family proteins are BAR domain containing proteins involved in organizing the plasma membrane. *S. pombe* encodes a sporulation-specific Pil1 family protein, Meu14, which has a specialized role in shaping the forespore membrane during sporulation. The functional analog of Meu14 in *S. cerevisiae* is Ssp1. While Ssp1 has no primary sequence homology to Pil1 or Meu14, AlphaFold predicts that it contains a Pil1-related BAR domain. Consistent with this structural prediction, mutation of residues in the putative lipid binding face of Ssp1 or in a residue implicated in multimerization disrupt sporulation. Characterization of the mutant proteins indicates that the BAR domain is necessary for recruitment of Ssp1 to the highly curved leading edge of the prospore membrane and multimerization of Ssp1 at that location is required for assembly of the leading edge complex. The distribution of Pil1 family proteins across an evolutionary tree of *Ascomycetes* reveals that Meu14 and Ssp1 arose independently in the lineages leading to *S. pombe* and *S. cerevisiae*, respectively.

## 1. Introduction

The Bin/Amphiphysin/Rvs (BAR) domain is a structural motif found in different membrane-associated proteins [1]. This domain is formed by dimerization of helical bundles that creates a concave or convex membrane binding surface that can either induce or sense curvature in a membrane [2]. Different subfamilies of BAR domains include the N-BAR and F-BAR proteins, which have concave binding surfaces, and the I-BAR proteins in which the membrane interaction surface is convex [1].

Within fungi, Pil1 proteins are a ubiquitous family of N-BAR containing proteins [3,4]. Oligomers of Pil1 proteins bind to plasma membranes through interaction with the lipid phosphatidylinositol 4,5 bisphosphate (PI4,5P2) [4,5]. This binding creates and stabilizes trench-like invaginations of the plasma membrane called eisosomes [6,7]. Eisosomes sequester specific proteins and lipids and are implicated in a variety of plasma membrane processes including regulation of endocytosis, stress response, and lipid homeostasis [8,9,10,11].

In *Saccharomyces cerevisiae* there are two Pil1 family proteins, Pil1 and its paralog Lsp1, with overlapping functions in eisosome assembly [4,5]. The fission yeast *Schizosaccharomyces pombe* similarly has paralogous Pil1 family members, termed Pil1 and Pil2, though these function independently to generate eisosomes at different stages of the life cycle [12].

*Ascomycete* yeasts are defined by the formation of ascospores. This process requires the fusion of secretory vesicles within the cytoplasm to create membrane compartments [13]. These compartments, termed prospore membranes in *S. cerevisiae*, expand as flattened, curved sheets to engulf the haploid nuclei formed by meiosis [14,15]. Eventually, the ends of this sheet meet and fuse resulting in capture of the nucleus within a double membrane [15,16]. Ascospore formation is similar throughout the ascomycetes [17,18,19,20]. For instance, in *S. pombe* ascospores form through the generation of analogous compartments termed forespore membranes that expand, engulf nuclei, and close to create spores [21,22].

In both *S. pombe* and *S. cerevisiae*, the leading edge protein complex (LEP) forms a ring at the lip of the growing prospore membrane and the LEP is essential to guide proper membrane expansion [23,24,25,26,27]. In *S. pombe*, the core component of the LEP is the Meu14 protein, a third Pil1 paralog in this organism [27]. In the absence of *meu14*^+^, spore formation is blocked [27]. The core LEP component in *S. cerevisiae* is Ssp1, which provides a platform upon which other proteins of the LEP assemble [24,25,26]. Similar to Meu14, Ssp1 localizes to the lip of the prospore membrane and *SSP1* is essential for sporulation [25]. Although *meu14*^+^ and *SSP1* exhibit analogous functions in prospore formation, no primary sequence conservation has been observed between the two proteins. However, as reported here, when the Ssp1 protein structure determined by AlphaFold was used to search for similar structures in other proteins, a Pil1-related BAR domain was discovered in the central region of Ssp1. This observation raises the intriguing possibility that the BAR domains of both Meu14 and Ssp1 function by binding to the highly curved lip of the growing prospore membrane. Point mutations in the Ssp1 BAR domain, predicted by the Alphafold model to interfere with membrane binding or oligomerization support the structural predictions. Analysis of the distribution of Pil1 family proteins across the *Ascomycetes* tree reveals that Meu14 and Ssp1 evolved independently in lineages leading to *S. pombe* and *S. cerevisiae*.

## 2. Materials and Methods

### 2.1. Strains and Media

Unless otherwise noted, standard growth and sporulation media were used [28]. All strains used are in the SK-1 background and their genotypes are listed in Table 1. The *SSP1* point mutations in strains AN1159 to AN1164 were introduced by two-step gene replacement [29]. First, *URA3*-integrating plasmids carrying different *SSP1* alleles were targeted to the promoter region upstream of *ssp1∆::kanMX6* by digestion with MscI before transformation. Ura+ transformants were selected, patched onto YPD plates, and then transferred to plates containing 5-fluoro-orotic acid (5-FOA) to select isolates that had lost the integrating plasmid, leaving either the *ssp1∆::kanMX6* or *ssp1-x* allele behind [29]. Individual 5-FOA resistant colonies were then screened for loss of geneticin resistance, indicating that the *ssp1∆::kanMX6* allele has been replaced by the point mutation, and the presence of the mutant allele was confirmed by PCR and sequencing.

### 2.2. Plasmids

Plasmids used in this study are listed in Table 2. To construct *URA3* integrating plasmids carrying the different *SSP1* point mutants, the *SSP1* coding region and 500bp of upstream and downstream sequence were amplified by PCR and inserted into SacI-KpnI cut pRS306 using the NEBuilder HiFI DNA Assembly Master Mix (hereafter referred to as Gibson Assembly) (New England Biolabs, Cat. # E2621) to create pRS306-SSP1. This plasmid was then used as a template to construct the different *SSP1* point mutations. Overlapping oligos carrying the specific mutations as well as overlapping oligos in the Ampicillin resistance gene were used to generate two PCR products. These products were then combined using Gibson Assembly to create pRS306-SSP1-AA (carrying the K185A, R186A mutations), pRS306 SSP1-AAA (K276A, K277A, K280), and pRS306-SSP1-R260A. The plasmids pRS314-SSP1-AA-YFP, pRS314-SSP1-AAA-YFP, and pRS314-SSP1-R260A-YFP were constructed using the same strategy and oligonucleotides, but pRS314-SSP1-YFP was used as the template. To generate sporulation-induced *PIL1-GFP*, the *PIL1* ORF was first amplified from genomic DNA, digested with SpeI and HindIII and ligated with similarly digested pGFP-C-FUS. The *PIL1-GFP* fusion was then isolated from this construct by SpeI/KpnI digestion and ligated into a similarly digested version of pRS424 carrying the *SPO20* promoter region, creating pRS424-P_SPO20_-PIL1-GFP. Sporulation-induced *meu14-GFP* was generated by amplifying the second exon of *meu14* encoding amino acids 28-335 (including the entire BAR domain) from *S. pombe* genomic DNA and cloning the product into EcoRV cut pRS313 [31]. The primers used introduce an ATG codon at the 5′ end of the coding region. This *meu14* coding region, lacking the N-terminal 27 amino acids was then cloned from this construct as a BamHI/ClaI fragment into similarly digested pRS424-P_SPO20_-GFP (a gift of H. Nakanishi).

### 2.3. Alphafold Predictions and Phylogenetic Analysis

Alphafold predictions of full length Ssp1 and the Ssp1 BAR domain alone (amino acids 181–360) were generated using the Alphafold 2 algorithm available at Google Colab (https://colab.research.google.com/github/sokrypton/ColabFold/blob/main/AlphaFold2.ipynb accessed on 3 January 2023). To examine the distribution of Pil1 family proteins in *Ascomycetes*, species representative of different branches of the *Ascomycete* tree were selected [36]. *S. cerevisiae* Pil1, *A. nidulans* PilB, *S.pombe* Meu14, and *S. cerevisiae* Ssp1 were separately used as queries in BLASTP searches (https://https://blast.ncbi.nlm.nih.gov/Blast.cgi?PROGRAM=blastp&PAGE_TYPE=BlastSearch&LINK_LOC=blasthome accessed 15 March 2023) of each of the selected proteomes. Reciprocal best hit criteria were used to assign orthology.

### 2.4. Microsocopy

For the fluorescence microscopy experiments, cells carrying the appropriate plasmids were patched onto selective medium, grown overnight at 30°, and then replica plated to sporulation (SPO) medium. SPO plates were incubated at 30° overnight and then cells were transferred to slides for imaging. Images were collected on a Zeiss Imager using Zeiss Zen 3.0 software and Adobe Photoshop and Adobe Illustrator to prepare the figures.

## 3. Results

### 3.1. Ssp1 Contains a Predicted BAR Domain with Strong Similarity to Pil1

**Figure 1:**
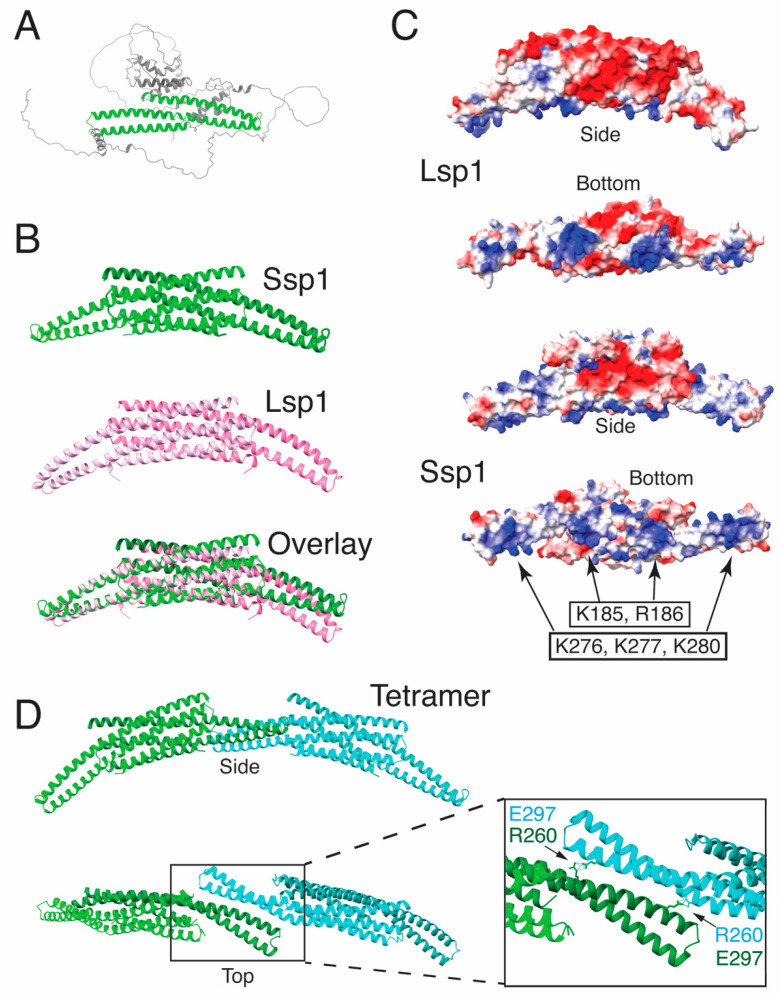
Ssp1 contains a predicted BAR domain. (**A**) AlphaFold prediction of the structure of *S. cerevisiae* Ssp1. The helices highlighted in green form the potential BAR domain. (**B**) A dimer of the predicted BAR domain of Ssp1 (aa 180-380) assembled using AlphaFold multimer is shown in green [41]. In pink is the BAR domain of Lsp1 as determined by X-ray crystallography [4]. (**C**) Surface electrostatics of the Lsp1 and Ssp1 BAR domains. Red indicates negative and blue indicates positive charges. Both proteins display four patches of positive charges across the concave (bottom) surface of BAR domain. The positions of residues mutated in *ssp1-AA* (K185, R186) and *ssp1-AAA* (K276, K277, K280) are indicated by the arrows. (**D**) Predicted arrangement of Ssp1 BAR multimers. Four copies of the Ssp1 BAR domain were assembled with AlphaFold multimer resulting in a prediction of a pair of dimers, one shown in green and one in blue. The inset at the right shows the dimer-dimer interface, stabilized by salt bridges between R260 and E297 in adjacent Ssp1 monomers.

Iterative BLAST searches starting with the *S. cerevisiae* Ssp1 identified orthologs in other yeasts, but no homology to any known proteins. The AlphaFfold predicted structure of Ssp1 was used as the starting query for a FOLDSEEK search to look for structural homologs [37,38,39]. This search identified the *Candida albicans* ortholog of Ssp1 (orf19.416) but also identified, at a similar level of confidence, Pil1 family proteins from *C. albicans*, *S. pombe*, and *S. cerevisiae*. The region of structural homology corresponded to the BAR domain of Pil1. The AlphaFold model of Ssp1 includes three long, overlapping α-helices that are arranged similarly as in known BAR domains (Figure 1A). BAR domains form dimers to create curved, membrane-binding surfaces [40]. When two copies of the putative BAR domain of Ssp1 (aa 180–360) were modeled together using AlphaFold Multimer [41], the algorithm predicted a dimer with the same length and arrangement of α-helices as in the crystal structure of the Lsp1 BAR domain (Figure 1B) [4]. Overlay of the structures revealed that the Lsp1 BAR domain is slightly more concave than that of Ssp1 (Figure 1B). The Lsp1 BAR domain binds membranes through basic residues on its concave surface [4,5]. Examination of the charge distribution on the concave surface of the Ssp1 dimer revealed a pattern of four basic patches (arrows in Figure 1C) distributed similarly to the basic patches on the Lsp1 BAR domain. The Ssp1 BAR domain may therefore also bind lipids.

AlphaFold multimer was then used to examine higher order oligomerization of the Ssp1 BAR domain. When four separate Ssp1 BAR domain sequences were input, AlphaFold predicted a tetramer consisting of a slightly overlapped alignment of dimers creating an extended and more deeply curved lipid binding surface (Figure 1D). This structure is consistent with the proposed pattern of multimerization of the Lsp1 protein [42]. In sum, the structural homology and AlphaFold modeling suggest that Ssp1 is a member of the Pil1 BAR domain family.

### 3.2. The AlphaFold Model for the Ssp1 BAR Domain Predicts Amino Acids Critical for Function

As a test of the AlphaFold model, the effects of mutations that are predicted to disrupt either the basic, membrane-binding surface of Ssp1 or the ability of the BAR domain to form higher order multimers were examined. Each monomer contains two clusters of positively charged residues, K185, R186 and K276, K277, K280, resulting in four basic patches on the concave surface in the dimer (Figure 1C). The amino acids in each cluster were separately changed to alanine to create two alleles of *SSP1*, *ssp1-K185A*,*R186A* (hereafter *ssp1-AA*) and *ssp1-R276A*, *K277A*, *K280A* (hereafter, *ssp1-AAA*). Additionally, the multimerization of Ssp1 BAR dimers is predicted to be stabilized by salt bridges between residues R260 and E297 on adjoining dimers (Figure 1D). An allele that is predicted to disrupt this interaction, *ssp1-R260A*, was also constructed. These three alleles are collectively described as BAR domain mutants.

Diploids homozygous for *ssp1-AA*, *ssp1-AAA* or *ssp1-R260* were examined for a variety of phenotypes. Spore formation was completely blocked by all three mutants and this defect was rescued by reintroduction of *SSP1* on an integrating plasmid (Table 3).

*SSP1* is not only required for proper prospore membrane growth but its timely removal is necessary to allow prospore membrane closure and spore development. Therefore, the lack of sporulation could be due either to a loss of Ssp1 function or the failure to shut off Ssp1. These two possibilities can be distinguished by looking at prospore membrane size in the BAR domain mutants. In *ssp1*∆ cells prospore membranes are generally smaller and abnormally shaped [25]. In contrast, when Ssp1 is not removed from the leading edge at the end of meiosis II in *ssp1* C-terminal truncations or in an *ama1*∆ mutant, the membranes grow excessively large [43,44].

**Figure 2:**
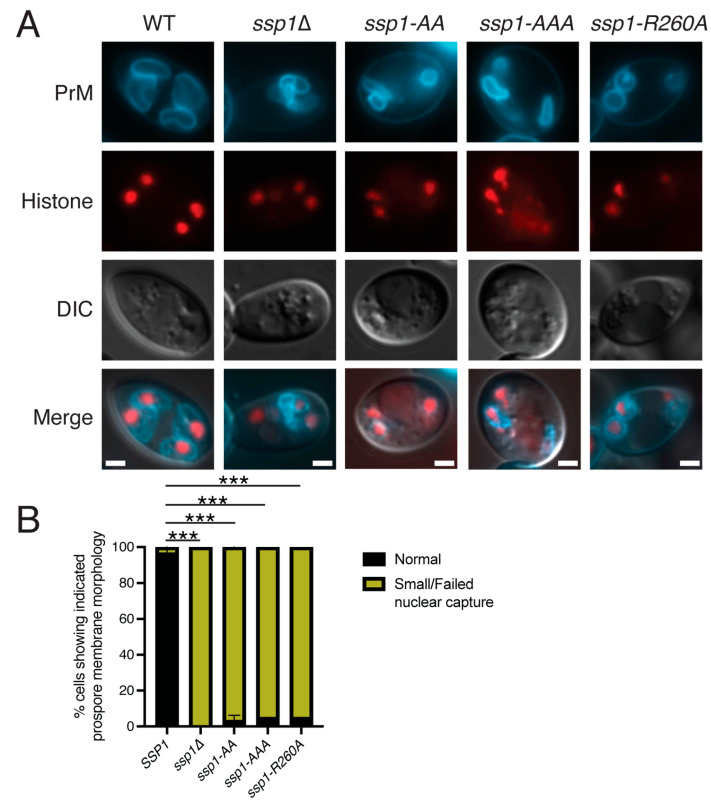
Prospore membrane growth is abnormal in *ssp1* point mutants. (**A**) Wild-type (AN120), *ssp1*∆ (NY551), *ssp1-AA* (AN618), *ssp1-AAA* (AN617), and *ssp1-R260A* (AN619) strains carrying plasmids expressing the fluorescent histone HTB1-mOrange2 and the prospore membrane marker Spo20^51-91^-mTagBFP. Strains were sporulated, post-MII cells identified by the histone fluorescence and the prospore membrane morphology in these cells examined. Scale bars = 1 micron. (**B**) Histogram of distribution of prospore membrane morphologies seen in each strain. Asterisks indicate that the difference in the appearance of normal prospore membranes between wild-type and the different mutants is significant (*** *p* < 0.0001) by Chi-squared test. A total of 60 cells were scored over three separate experiments for each plasmid. Error bars indicate one standard deviation.

Prospore membranes were visualized in BAR domain mutant diploids using a blue fluorescent marker for the prospore membrane, mTagBFP-Spo20^51-91^ [35]. Cells in meiosis II were identified using mOrange tagged Histone Htb1 [34]. Prospore membranes were examined specifically in late MII cells that were identified by the presence cells of four Htb1-mOrange fluorescent nuclei (Figure 2A). Prospore membranes in wild-type cells appeared as large ovals or rings, each ring encircling a nucleus marked by Htb1-mOrange fluorescence. By contrast, in *ssp1*∆ cells, the prospore membranes appeared smaller, misshapen, and sometimes failed to encapsulate nuclei. All three *ssp1* BAR domain mutants, exhibited small and/or misshapen prospore membranes similar to the *ssp1*∆ deletion (Figure 2). Therefore, all three mutants behave as loss of function mutants.

### 3.3. The Basic Patches on Ssp1 Are Required for Localization to the Leading Edge, but Not Prospore Membrane Association

The basic patches in the BAR domain of Pil1/Lsp1 are required for membrane binding [4]. If the domain in Ssp1 predicted to have structural homology with Lsp1 functions as a BAR domain, then mutation of the basic patches in this domain should disrupt prospore membrane binding. To examine the localization of the mutant proteins each mutation was introduced into an *SSP1-YFP* (Yellow Fluorescent Protein) allele carried on a replicating plasmid and transformed into an *ssp1*∆ mTagBFP-Spo20^51-91^ Htb1-mOrange diploid so that the *ssp1-YFP* alleles were the only source of Ssp1 protein [24].

**Figure 3:**
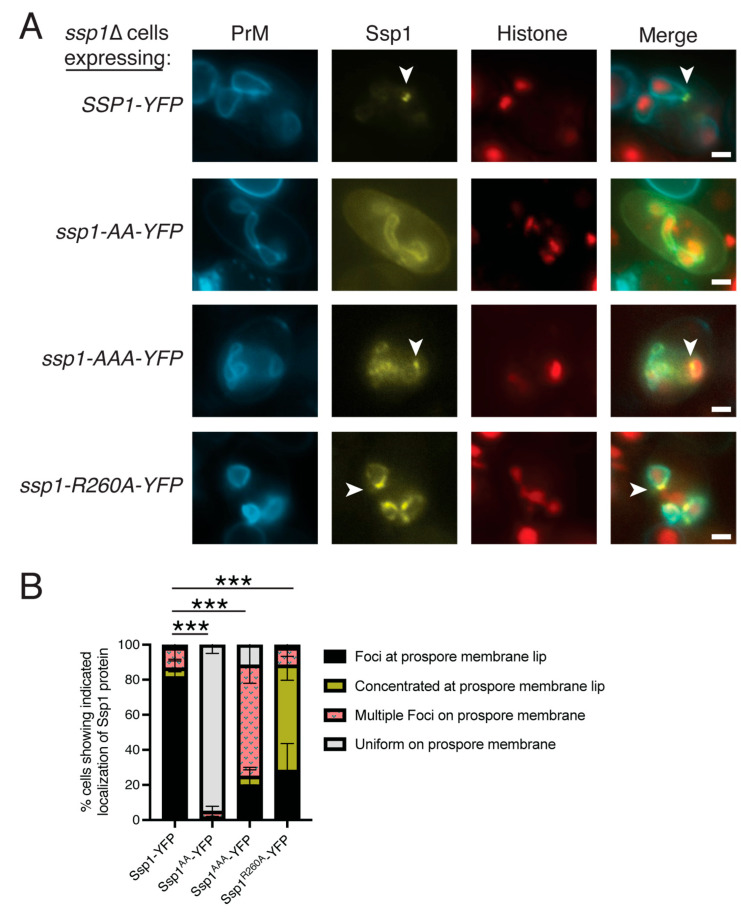
Abnormal localization of the Ssp1 BAR domain mutant proteins. (**A**) An *ssp1*∆ strain (NY551) was transformed with CEN plasmids expressing C-terminal YFP fusions to wild type or mutant Ssp1 proteins. The cells also carried HTB1-mOrange2 and Spo20^51-91^-mTagBFP as nuclear and prospore membrane markers, respectively. Cells were sporulated and MII cells identified by the histone fluorescence pattern. Arrowheads highlight examples of Ssp1 fluorescence at the lip of the prospore membrane. Scale bars = 1 micron. (**B**) Histogram of distribution of localization patterns seen in each strain. Asterisks indicate that the difference in the appearance of Ssp1 foci at the prospore membrane lip between wild-type and the different mutants is significant (*** *p* < 0.0001) by Chi-squared test. A total of 60 cells were scored over three separate experiments for each plasmid. Error bars indicate one standard deviation.

The localization of the Ssp1-YFP fusion proteins was scored only in cells displaying fluorescence from all three markers. As previously reported, the wild-type Ssp1-YFP protein was found localized at the tips of the growing prospore membrane (80% of prospore membranes) (Figure 3A,B) [24]. By contrast, the Ssp1^AA^-YFP protein was uniformly distributed along the prospore membrane (95%) (Figure 3A,B). These membranes also appeared abnormal, consistent with the *ssp1-AA* phenotype (Figure 3A). Thus, mutation of the K185, R186 patch does not disrupt membrane binding, but disrupts the ability of Ssp1 to concentrate at the leading edge of the prospore membrane.

Unlike Ssp1^AA^-YFP, the Ssp1^AAA^-YFP pattern was mixed. The protein displayed uniform YFP fluorescence along the prospore membrane, as with Ssp1^AA^-YFP, in 15% of the cells, while another in 60% of the cells, Ssp1^AAA^-YFP was seen in multiple, discrete foci along the prospore membrane (Figure 3B). In the remaining 25% of the cells, Ssp1^AAA^-YFP was localized at the lip of the prospore membrane either as a single focus or as a broader patch similar to Ssp1-YFP. Different prospore membranes within the same cells sometimes displayed different localization patterns (Figure 3A). Consistent with the possibility that the normally localized Ssp1^AAA^-YFP is functional, 15% of *ssp1-AAA-YFP* cells sporulated (Table 3). Why the *ssp1-AAA-YFP* mutant expressed from a plasmid exhibits a leakier phenotype than the chromosomal *ssp1-AAA* homozygous diploid is unclear (Table 3), though it suggests that the basic patch created by K276, K277, K280 is not as essential as the K185, R186 patch. Though their effects vary in severity, both mutations on the basic surface of the protein affect localization to the leading edge of the prospore membrane without losing membrane association. In the context of the AlphaFold model (Figure 1), this suggests that the negative charges on the concave face of the BAR domain are important for binding to the highly curved leading edge, but not membrane association per se.

### 3.4. The Ssp1^R260A^ Protein Localizes to the Leading Edge

The AlphaFold model predicts that the R260A mutation is required for multimerization of Ssp1 but should not disrupt membrane binding as the basic patches are unaffected. The localization of Ssp1^R260A^-YFP protein appeared more like wild-type. There was a clear concentration at or near the prospore membrane lip in ~80% of the cells (Figure 3), though more often the localization appeared as a broader patch rather than as discrete foci as with Ssp1-YFP (Figure 3B). This indicates that, unlike the basic patch BAR domain mutants, Ssp1^R260A^ retains an affinity for the prospore membrane lip although it cannot support proper spore formation.

### 3.5. The BAR Domain Is Required for Leading Edge Complex Formation

**Figure 4:**
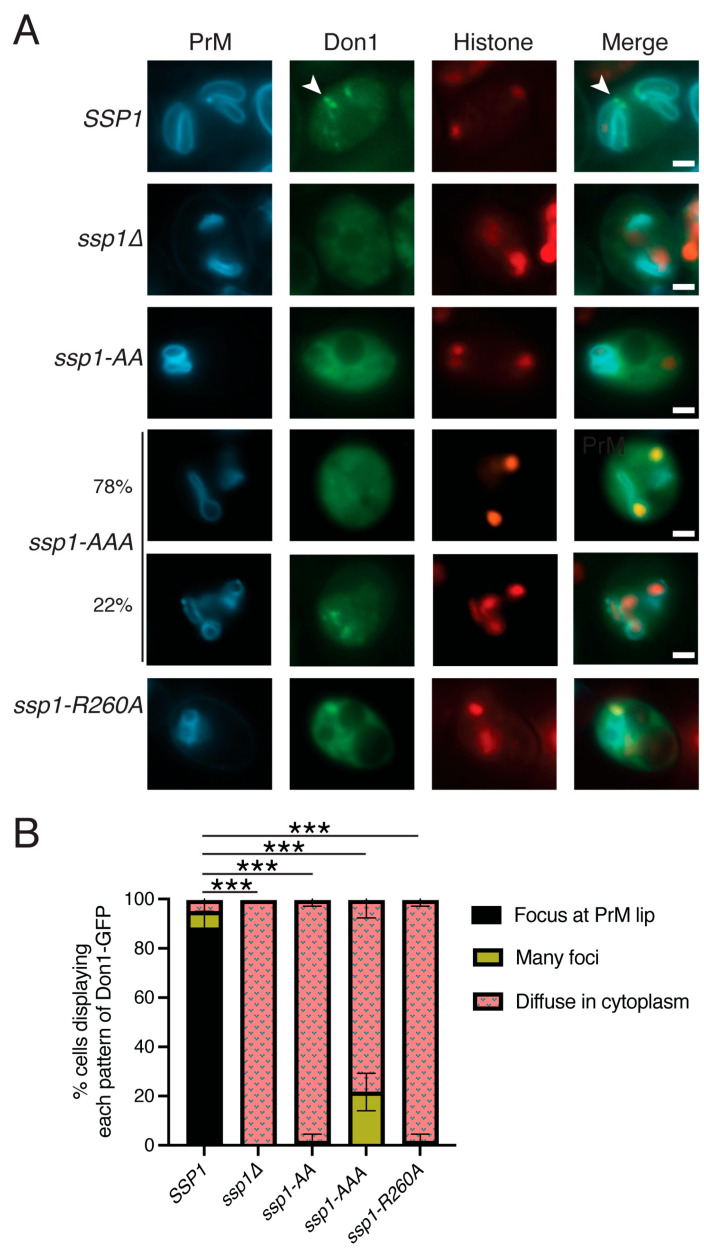
The leading edge complex does not assemble in the *ssp1* mutants. (**A**) Wild-type (AN120), *ssp1*∆ (NY551), *ssp1-AA* (AN618), *ssp1-AAA* (AN617), and *ssp1-R260A* (AN619) strains were transformed with plasmids expressing Don1-GFP as a marker for the leading edge complex, HTB1-mOrange2 and Spo20^51-91^-mTagBFP. Strains were sporulated, MII cells identified by the histone fluorescence and the localization of Don1-GFP examined. Arrowheads highlight an example of Don1 fluorescence at the leading edge of the prospore membrane. Scale bars = 1 micron. (**B**) Histogram of distribution of Don1 localization patterns seen in each strain. Asterisks indicate that the difference in the appearance of Don1 foci at the prospore membrane lip between wild-type and the different mutants is significant (*** *p* < 0.0001) by Chi-squared test. A total of 60 cells were scored over three separate experiments for each plasmid. Error bars indicate one standard deviation. “PrM” indicates prospore membrane.

*SSP1* is required for formation of the leading edge complex that includes Don1, Ady3 and Irc10 [24,25,26]. If the presence of Ssp1 on prospore membranes is sufficient to recruit Don1, then the localization of Don1 in *ssp1-AA* or *ssp1-AAA* cells should mirror the abnormal Ssp1-AA-YFP and Ssp1-AAA-YFP localization patterns. This idea was tested by introducing *DON1-GFP* into *SSP1*, *ssp1-AA*, and *ssp1-AAA* diploids carrying *HTB1-mOrange2* and *spo20^51-91^-mTagBFP*. As expected, Don1 localized to the leading edges of prospore membranes in *SSP1* cells (90% of prospore membranes scored), but was diffusely distributed throughout the cytoplasm in *ssp1*∆ cells (100%) (Figure 4A,B) [25]. Don1-GFP also appeared throughout the cytoplasm in *ssp1-AA* and the majority of *ssp1-AAA* cells (78%) (Figure 4) even though these mutant Ssp1 proteins are present on the prospore membrane (Figure 3). Therefore, the presence of Ssp1 is not sufficient to recruit Don1. A significant fraction (22%) of *ssp1-AAA* cells exhibited some localization of Don1 to the prospore membrane, consistent with the leaky nature of this allele. These results suggest that Ssp1 may be first recruited to leading edge via its BAR domain to create a platform on which the leading edge complex can assemble.

The Ssp1-R260A protein localized to leading edges of prospore membranes, but Don1 was still cytoplasmically localized in the *ssp1-R260A* mutant (100%) (Figure 3 and Figure 4). Therefore, the presence of Ssp1 at leading edges is not sufficient to recruit Don1 either. Given that the *ssp1-R260A* mutant is proposed to disrupt multimerization of Ssp1, Don1 interaction may only occur when the multimerized form of Ssp1 is present at the leading edge.

### 3.6. Evolution of the Pil1 Family in Ascomycetes

**Figure 5:**
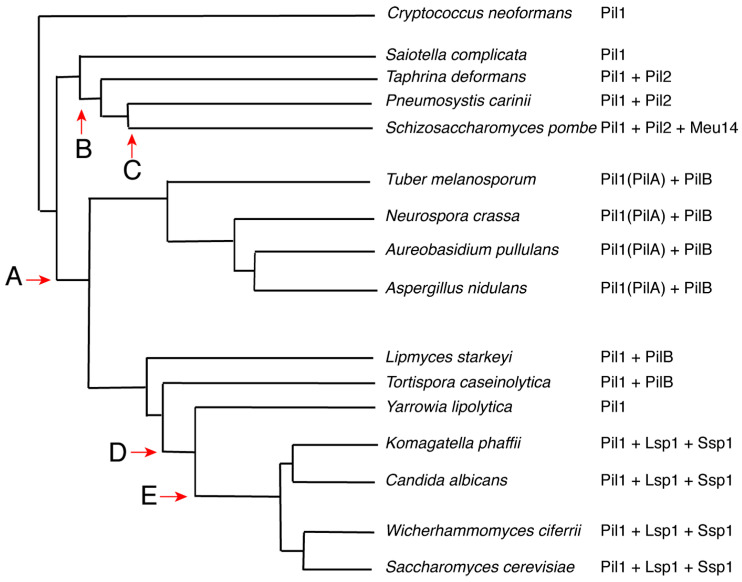
Evolution of the PIL1 family in *Ascomycetes*. The content of Pil1 BAR domain proteins was examined in the genomes of representative members of different ascomycete lineages. The branching pattern of the tree is based on [36]. The basidiomycete *C. neoformans* was used as an outgroup. The inferred positions of gene losses or duplications are indicated by the letters. A—Duplication to give rise to Pil B; B—Duplication to give rise to Pil2; C—Duplication to give rise to Meu14; D—Loss of PilB; E—Duplications to give rise to Lsp1 and Ssp1. Branch lengths are not to scale.

The ability of the AlphaFold model to successfully predict critical functional residues within Ssp1 strongly supports the idea that Ssp1 is a *bona fide* Pil1 BAR domain protein. To better understand the evolution of this protein family the content and distribution of Pil1 family proteins was examined across a phylogenetic tree of ascomycete species (Figure 5) [36]. Several representative species were selected in each branch of the ascomycetes; fission yeasts (archaeasocmycetes), hyphal fungi (sordariamycetes and pezizomycetes), and budding yeasts (saccharomycetes). The basidiomycete *C. neoformans* was selected as an outgroup. The complete proteome of each of the species was searched using known Pil1 BAR domain proteins as probes to identify the content of Pil1-related proteins in each species. The results reveal a complicated pattern of gene duplication and loss (Figure 5). The last common ancestor of the *Ascomycetes* is inferred to have had only a single, ancestral *PIL1* gene. After the divergence of the fission yeasts from the line leading to hyphal fungi and budding yeasts, there was a gene duplication in this latter lineage creating PilB (‘A’ in Figure 5). Separately, during the evolution of the fission yeasts there was a duplication of Pil1 giving rise to Pil2 and then a second duplication giving rise to Meu14 (‘B’ and ‘C’ in Figure 5). All hyphal fungi examined still carry Pil1 (referred to in these organisms as PilA) and PilB, however during the evolution of budding yeasts PilB was lost (‘D’ in Figure 5) and then after the divergence of *Yarrowia* two additional duplications occurred giving rise to Lsp1 and to Ssp1. This pattern of conservation indicates that, although both Meu14 and Ssp1 are sporulation-specific Pil1 family members, they evolved independently in their separate lineages.

### 3.7. Neither Pil1 nor Meu14 Localize to the Prospore Membrane

**Figure 6:**
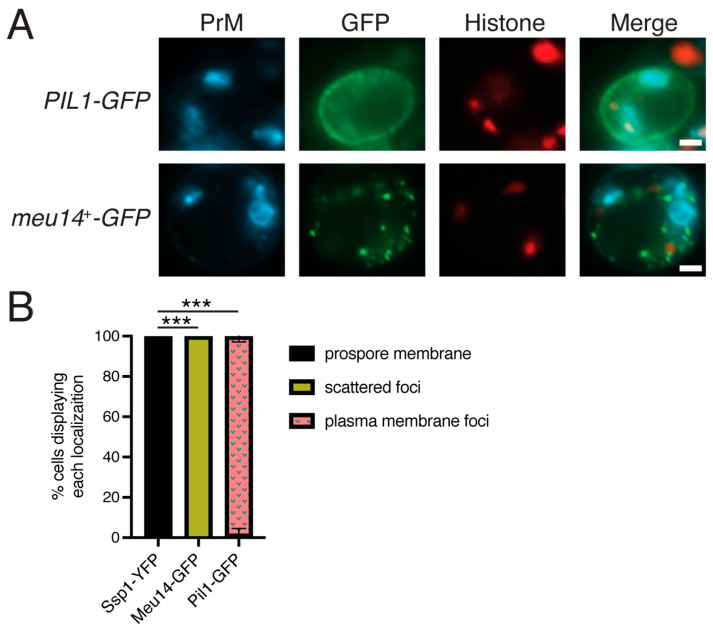
Pil1 and Meu14 cannot localize to the leading edge. (**A**) An *ssp1*∆ strain (NY551) was transformed with C-terminal GFP fusions to *Pil1* or *meu14*^+^ under control of the sporulation-induced *SPO20* promoter. The cells also carried HTB1-mOrange2 and Spo20^51-91^-mTagBFP as nuclear and prospore membrane markers, respectively. Cells were sporulated and MII cells identified by the histone fluorescence pattern. Scale bars = 1 micron. (**B**) Histogram of distribution of localization patterns seen in each strain. Asterisks indicate that the difference in the appearance of foci at the prospore membrane between Ssp1 and Pil1/Meu14 is significant (*** *p* < 0.0001) by Chi-squared test. (Ssp1 data are from Figure 3). A total of 60 cells were scored over three separate experiments for each plasmid. Error bars indicate one standard deviation.

Given the evolutionary relationships of Pil1, Meu14, and Ssp1 whether either of the first two proteins could potentially substitute for Ssp1 was examined. Fusions of GFP to either *S. cerevisiae PIL1* or *S. pombe meu14^+^* were placed under the control of the sporulation-induced *SPO20* promoter on plasmids, introduced into an *ssp1*∆ strain, and the protein localization was examined during meiosis II (Figure 6). Rather than localize to prospore membranes, Pil1-GFP was localized into foci, presumably eisosomes, along the plasma membrane (100% of cells scored). Meu14-GFP behaved differently, localizing in discrete puncta throughout the cytoplasm (100%). What this localization represents is not clear, but these puncta do not colocalize with a prospore membrane marker. Thus, the ability of Ssp1 to localize to the leading edge in *S. cerevisiae* is unique. Neither Pil1 nor Meu14 localize to the leading edge and they appear to have no affinity for the prospore membrane.

## 4. Discussion

### 4.1. SSP1 Encodes a BAR Domain Protein of the Pil1 Family

AlphaFold predicts a potential BAR domain within Ssp1 similar to that of Pil1. If this prediction is accurate, basic patches on the concave surface of the structure should be important for association of Ssp1 with the membrane and Ssp1 function. Indeed, mutation of either patch results in loss of function and failure to sporulate. Localization of the Ssp1^AA^ and Ssp1^AAA^ proteins reveals that they still associate with the prospore membrane, though they no longer concentrate at the leading edge. These results suggest the basic patches are required for binding to curved membranes rather than membranes per se. This result is reminiscent of Pil1 mutants in which a basic patch in the concave surface of the BAR domain is mutated. In that case, the mutant Pil1 can no longer associate with eisosomes but can still bind to the plasma membrane through a lipid binding site outside of the BAR domain [4,42]. Interestingly, Ssp1 has previously been shown to bind lipids through a basic patch N-terminal to the predicted BAR domain [44].

Unlike the first two BAR domain mutants, the third mutant designed from the Alphafold structure, Ssp1^R260A^, displayed a distribution similar to the wild-type protein. However, this allele also displayed a null phenotype and failed to recruit Don1, indicating that R260 is essential for function. Based on the AlphaFold structure, this residue is predicted to block multimerization of the BAR domain, suggesting that Ssp1 oligomers may be necessary for leading edge complex assembly. Further biochemical studies will be necessary to determine if this mutation actually alters multimerization of Ssp1. Nonetheless, the fact that all three mutants displayed phenotypes supports the AlphaFold prediction that Ssp1 is a BAR domain protein related to Pil1.

### 4.2. Ssp1 Behavior Is Distinct from Pil1/Lsp1

When Pil1 and Lsp1 bind to the plasma membrane they both induce and stabilize membrane curvature creating the eisosome, and in their absence eisosomes don’t form [6,7,42]. By contrast, the highly curved lip of the prospore membrane is present even in the absence of *SSP1* [25]. Rather than creating a curved membrane domain, Ssp1 appears to recognize the curved membrane region and play a role in promoting proper growth of the whole prospore membrane by keeping the mouth of the prospore membrane open [44].

How does Ssp1 recognize the lip of the prospore membrane? The observation that both Ssp1^AA^ and Ssp1^AAA^ proteins are delocalized indicates that the positive charges on the concave surface of the BAR domain are required for recognition. Neither Pil1 nor Meu14 can localize to the leading edge of the prospore membrane (Figure 6), indicating that the presence of a Pil1 family BAR domain alone is insufficient to recognize this curved region of the membrane.

Pil1 associates with the plasma membrane by binding to PI4,5P2 [4,5]. The inability of Pil1 to be recruited to the leading edge of the prospore membrane is consistent with studies showing that PI4,5P2 is found predominantly on the plasma membrane during meiosis, while the prospore membrane is relatively enriched in phosphatidylinositol-4-phosphate (PI4P) [44,45].

When Ssp1 is ectopically expressed in vegetative cells, the protein localizes to the plasma membrane and to secretory vesicles clustered under the plasma membrane [44]. Binding to the plasma membrane in this context is mediated by a PI4,5P2 binding site located outside of the BAR domain [44]. When an Ssp1 mutant lacking this PI4,5P2 binding site is expressed in vegetative cells, localization to the plasma membrane is lost, but localization to the secretory vesicles is unaffected. This indicates that the Ssp1 BAR domain does not have affinity for the plasma membrane. The secretory vesicles to which Ssp1 localizes in vegetative cells have a high curvature, similar to the lip of the prospore membrane. Additionally, these vesicles are enriched in PI4P, as is the prospore membrane [45,46]. It is possible that the combination of high membrane curvature and PI4P are what recruit Ssp1 to the leading edge.

### 4.3. Convergent Evolution of a Role for Pil1 Family Proteins in Prospore Membrane Assembly

Pil1 family proteins are a found throughout the fungi and the organization of eisosomes on plasma membranes is an important conserved role of this protein family [3,47]. This work reports a second shared function for Pil1 BAR domain proteins, guiding morphogenesis of the prospore membrane during ascospore formation. In both *S. pombe* and *S. cerevisiae*, specialized Pil1-family proteins have evolved for this function. Two alternative trajectories for this evolutionary path seem possible. First, it may be that the ancestral Pil1 functioned both in eisosome formation and in prospore membrane formation. In this instance, both Meu14 and Ssp1 could have arisen from events where there was a duplication of a *PIL1* gene followed by specialization in which one copy retained eisosome function and the other retained sporulation function. Alternatively, ancestral Pil1 may have had only a role in eisosome function and, after duplication, Meu14/Ssp1 could have acquired a novel role in prospore membrane growth.

These two possibilities can be distinguished by examining the role of PilA and/or PilB in ascospore formation in filamentous ascomycetes (Figure 5). If ancestral Pil1 is important for prospore membrane growth, then either PilA or PilB would be expected to retain that role in sporulation. Helpfully, the localization of PilA-GFP and PilB-GFP during the sexual cycle of *Aspergillus nidulans* has been reported [48]. While both proteins can be seen in mature ascospores, neither protein is visible during ascospore formation. Moreover, neither pil*A* nor *pilB* mutants have any effect on ascospore formation [48]. These observations indicate that Pil1 family proteins are not involved in prospore membrane growth in *Aspergillus*. This finding suggests that Meu14 and Ssp1 independently acquired the identical role in sporulation in the lineages leading to *S. pombe* and *S. cerevisiae*, a remarkable instance of convergent evolution.

## Figures and Tables

**Table 1:** Strains used in this study.

Strain	Genotype	Source
AN117-4B	*MATα ura3 his3∆SK leu2 trp1::hisG lys2 arg4-NspI rme1∆::LEU2 ho∆::LYS2*	[30]
AN117-16D	*MAT***a** *ura3 his3∆SK leu2 trp1::hisG lys2 ho∆::LYS2*	[30]
NY66	same as AN117-4B except *ssp1∆::kanMX6*	[24]
NY67	same as AN117-16D, except *ssp1∆::kanMX6*	[24]
AN1159	same as AN117-4B, except *ssp1-K276A K277A K280A*	this study
AN1160	same as AN117-16D, except *ssp1-K276A K277A K280A*	this study
AN1161	same as AN117-4B, except *ssp1-K185A R186A*	this study
AN1162	same as AN117-16D, except *ssp1-K185A R186A*	this study
AN1163	same as AN117-16D, except *ssp1-R260A*	this study
AN1164	same as AN117-4B, except *ssp1-R260A*	this study
AN120	*MATα ura3 his3∆SK leu2 trp1::hisG lys2 arg4-NspI rme1∆::LEU2 ho∆::LYS2* *MAT***a** *ura3 his3∆SK leu2 trp1::hisG lys2 ARG4 RME1 ho∆::LYS2*	[30]
NY551	same as AN120 except *ssp1∆::kanMX6*	[24]
AN617	same as AN120 except *ssp1-K276A K277A K280A*	this study
AN618	same as AN120 except *ssp1-K185A R186A*	this study
AN619	same as AN120 except *ssp1-R260A*	this study

**Table 2:** Plasmids used in this study.

Plasmid	Relevant Features	Source
pRS306	*URA3*	[31]
pRS314	*TRP1 CEN ARS*	[31]
pRS313	*HIS3 CEN ARS*	[31]
pRS424	2µ *TRP1*	[32]
pRS426	2µ *URA3*	[32]
pGFP-C-FUS	*GFP*	[33]
pRS424-P_SPO20_-GFP	2µ *TRP1 P_SPO20_*	H. Nakanishi
pRS424-P_SPO20_	2µ *TRP1 P_SPO20_ GFP*	H. Nakanishi
pRS306-SSP1	*URA3 SSP1*	This study
pRS306-SSP1-AA	*URA3*	this study
pRS306-SSP1-AAA	*URA3*	this study
pRS306-SSP1-R260A	*URA3*	this study
pRS314-SSP1-YFP	*TRP1 CEN ARS SSP1-YFP*	[24]
pRS314-SSP1-AA-YFP	*TRP1 CEN ARS ssp1-K185A R186A-YFP*	this study
pRS314-SSP1-AAA-YFP	*TRP1 CEN ARS* *ssp1-K276A K277A K280A-YFP*	this study
pRS314-SSP1-R260A-YFP	*TRP1 CEN ARS ssp1-R260A-YFP*	this study
pRS313-HTB1-mOrange2	*HIS3 CEN ARS HTB1-mOrange2*	[34]
pRS426-Spo20^51-91^-mTagBFP	*URA3 2*µ *spo20^51-91^-mTagBFP*	[35]
pRS424-P_SPO20_-Pil1-GFP	*TRP1 2*µ *P_SPO20_-PIL1-GFP*	this study
pRS424-P_SPO20_-Meu14-GFP	*TRP1* 2µ *P_SPO20_-meu14^+^-GFP*	this study
pSB9	*TRP1* 2µ *DON1-GFP*	[24]

**Table 3:** Sporulation efficiencies of *ssp1* BAR domain mutants.

Genotype ^1^	Gene Expressed from Plasmid ^2^	% Sporulation (±SD) ^3^
*SSP1*	-	73.8 (±4.9)
*ssp1*∆	-	0 (±0)
*ssp1-AA*	-	0 (±0)
*ssp1-AA*	*SSP1*	68 (±6.5)
*ssp1-AAA*	-	0 (±0)
*ssp1-AAA*	*SSP1*	70.3 (±8)
*ssp1-R260A*	-	0 (±0)
*ssp1-R260A*	*SSP1*	71 (±4.4)
*ssp1*∆	*SSP1-YFP*	32.5 (±6.6)
*ssp1*∆	*ssp1-AA-YFP*	0 (±0)
*ssp1*∆	*ssp1-AAA-YFP*	15.5 (±1.8)
*ssp1*∆	*ssp1-R260A-YFP*	0 (±0)

^1^ Strains used, AN120 (*SSP1*), NY551 (*ssp1*∆), AN618 (*ssp1-AA*), AN617 (*ssp1-AAA*), and AN619 (*ssp1-R260*). ^2^ Wild-type *SSP1* was introduced in the integrating vector pRS306; YFP fusions were expressed from the CEN vector pRS314. ^3^ Average of three independent experiments. 200 cells scored in each experiment.

## Data Availability

The original contributions presented in this study are included in the article. Further inquiries can be directed to the corresponding author.

## Abbreviations

The following abbreviations are used in this manuscript:

BAR	Bin/Amphiphysin/Rvs
PrM	Prospore Membrane
GFP	Green Fluorescent Protein
YFP	Yellow Fluorescent Protein
BFP	Blue Fluorescent Protein
